# Prior immunological memory to pertussis toxin affects the avidity development of anti-PT IgG antibodies after acellular pertussis booster vaccination

**DOI:** 10.1080/22221751.2025.2547720

**Published:** 2025-08-13

**Authors:** Aapo Knuutila, Niina Ahvenainen, Alex-Mikael Barkoff, Jussi Mertsola, Pieter van Gageldonk, Annemarie Buisman, Marta Valente Pinto, Dominic Kelly, Qiushui He

**Affiliations:** a Institute of Biomedicine, University of Turku, Turku, Finland; b Department of Life Technologies, University of Turku, Turku, Finland; c Department of Pediatric and Adolescent Medicine, Turku University Hospital, Turku, Finland; d National Institute for Public Health and the Environment, Centre for Infectious Disease Control, Bilthoven, The Netherlands; e Department of Paediatrics, Oxford Vaccine Group, University of Oxford, Oxford, UK; f Oxford University Hospitals NHS Foundation Trust, Oxford, UK; g InFLAMES Research Flagship Center, University of Turku, Turku, Finland

**Keywords:** Pertussis, pertussis toxin, IgG antibody, avidity, ELISA, vaccination

## Abstract

Acellular pertussis vaccines are used in many countries. Since the quantity of antibodies after vaccination wanes quickly, to study functional antibody properties is important for evaluating long-lasting protection. Additionally, substantial variation in the quantity and quality of antibodies exists after vaccination in different age groups. The avidity of antibodies to pertussis toxin (PT) after Tdap3-IPV booster vaccination was studied in children, adolescents, young adults, and older adults. Serum samples (*N = *365) were collected before, one month, and one year after vaccination in Finland, the Netherlands, and the United Kingdom. The samples were diluted to equal anti-PT IgG concentrations, and avidity was measured utilizing urea as a chaotropic agent. Although concentrations of anti-PT IgG at baseline were similar between the countries, avidity was higher in the Netherlands and United Kingdom. Despite increased anti-PT IgG concentrations in participants after vaccination, an increase in avidity was noted mainly among participants with low pre-vaccine avidity. Avidity was significantly lower in older adults in comparison to children (*p* < 0.01) and adolescents (*p* = 0.03) in Finnish participants one month after vaccination. Avidity after booster was influenced by the initial level of avidity, which could be linked to vaccination background, age, and prior disease exposure. The development of avidity from one month after vaccination to a year after was highly individual, with some participants having either a decrease, an increase or a stagnant level of avidity. This emphasizes that long-term follow-up of avidity is essential. Booster vaccination seems particularly beneficial to individuals with low antibody avidity before vaccination.

## Introduction

Pertussis is a vaccine-preventable disease, and after the introduction of vaccines in the 1950s, child morbidity and mortality rates have decreased significantly. Pertussis immunization is typically combined with tetanus and diphtheria vaccines (Tdap or DTwP, acellular and whole-cell pertussis vaccines, respectively) [1]. In 2023, the WHO estimated that 84% of children globally were immunized with a three-dose primary series [2]. Vaccination programmes for pertussis booster vaccinations vary widely between countries. For example, Tdap boosters are given at different ages and to different target populations, such as pregnant women, military conscripts, healthcare workers, and older adults. Despite extensive vaccination programmes, during recent decades pertussis has had a resurgence in many countries with high vaccination coverage [3–5]. Waning immunity and decay of antibody concentrations after vaccination have been speculated as key factors in this resurgence [6,7].

Although higher antibody concentrations to PT have been shown to correlate in general with better clinical protection [8–11], some individuals with high antibody concentrations after Tdap vaccination still contract typical pertussis disease [9], which emphasizes the importance of determining the functional characteristics of antibodies for establishing correlates for protection [12].

Antibodies differ in affinity and in the specificities of the epitopes on proteins of the targeted microbial pathogen. The avidity of antibodies describes the combined binding strength with multivalent antigens. The maturation of antibodies and an increase in affinity and avidity result from somatic hypermutation as an efficient, antigen-driven selection process [13–15]. Avidity maturation increases as time elapses after antigen exposure and with the age of the host [16–18] as only high-affinity antibodies are selected within the germinal centres [19,20]. During the acute phase of an infection or immediately after vaccination, low-affinity antibodies predominate, but over time high-affinity antibodies become more prevalent [21]. Increased antibody avidity can be considered as a marker of B cell maturation and improved memory against an antigen [17,18] and thereby able to induce long-term protection against disease [22]. For example, increased avidity following HiB conjugate vaccination is a possible factor in explaining why the observed protective efficacy can be better even with lower antibody concentrations [18,23,24]. Similarly for pertussis, high antibody concentrations do not guarantee protection, and some individuals are protected despite lower concentrations [25,26].

**Figure 1.**
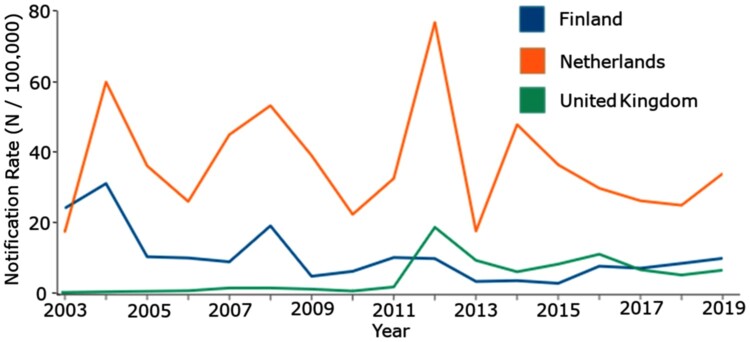
Laboratory confirmed pertussis notification rates in Finland, the Netherlands, and the United Kingdom at the time of the study, between October 2017 and January 2019. The figure is reproduced from disease data from the ECDC Surveillance Atlas [30].

In a recent Tdap booster trial, the IgG antibody concentrations, pertussis toxin (PT) neutralizing antibody (PTNA) capability, and frequencies of memory B cells were reported after acellular pertussis (aP) booster vaccination in four age groups with different vaccine priming backgrounds, in Finland, the Netherlands, and the United Kingdom (UK) [7,27,28]. Notably, the prevalence of the disease varies between the countries (Figure 1). The pertussis vaccination coverage at age one during the study period was 94% in the Netherlands and the UK, and 99% in Finland [29]. A subpopulation of this study is now further analysed to determine the existing antibody avidity before the Tdap booster vaccination, and the development of avidity at one month and one year after booster vaccination. Furthermore, the possible interplay between avidity and the other vaccination responses was evaluated, as mentioned above.

## Materials and methods

### Study approval

The “Booster against pertussis” clinical study was registered at the European Clinical Trials database (2016-003678-42) and was approved by the Medical Research Ethics Committees of respective countries [7]. Participants provided written informed consent.

### Study design

Participants of the study cohort (*N* = 365) (Table 1) received a booster dose of a Tdap3-IPV vaccine (Boostrix™-IPV – GlaxoSmithKline (GSK), Wavre, Belgium) between 2017–2019 in Finland, the Netherlands, and the UK. Serum samples were analysed from pre-booster, one month after, and one year after booster vaccination. Only samples with higher than 2.5 international units per mL (IU mL^−1^) of anti-PT IgG-antibodies were included, measured either by ELISA [16] or bead-based Luminex multiplex immunoassayas previously described [7,31].

**Table 1.** Study cohorts.

	Country^a^	Age (Mean yr)	No. of samples D0, D28, D365	No. of Female/Male	Primary vaccination^b^	Booster vaccination^b^, age	Anti-PT IgG (Mean IU mL^−1^) (D0, D28, D365)^b^
Children	FI NL UK	9.0 8.5 9.2	13, 34, 33 28, 36, 35 27, 34, 34	17/17 18/18 16/18	aP	aP, 4 years aP, 4 years aP, 3.3 years	30, 276, 60 31, 181, 49 24, 148, 57
Adolescents (aP)	FI NL UK	12.5 12.4 12.8	6, 17, 15 23, 25, 25 34, 35, 35	6/11 17/8 18/17	aP	aP, 4 years aP, 4 years aP, 3.3 years	47, 237, 71 35, 166, 53 20, 188, 65
Adolescents (wP)	FI NL	15.0 14.8	4, 18, 17 15, 23, 22	11/7 14/9	wP	aP, 2^c^ and 6 years aP, 4 years	51, 266, 87 38, 282, 80
Young adults	FI NL UK	30.2 28.6 26.1	5, 24, 24 11, 23, 20 12, 24, 21	20/4 9/14 16/8	wP	n/A	46, 134, 39 20, 258, 61 6, 173, 43
Older adults	FI NL UK	64.2 65.9 65.7	11, 23, 22 22, 24, 24 19, 25, 24	19/4 13/11 13/12	wP/unknown	n/A	39, 270, 114 28, 327, 72 11, 120, 52

^a^ FI = Finland, NL = The Netherlands, UK = The United Kingdom.

^b^ Detailed vaccine compositions, schedules, and anti-PT IgG concentrations are described in [7].

^c^ One participant was boosted by whole-cell pertussis vaccine (wP) at two years of age.

### Avidity assays

A standardized assay for studying avidity against pertussis antigens has not been established [26,32]. Avidity-index (AI) is most commonly used to describes the percentage of antibodies resisting elution in the presence of a chaotropic agent. For this study, 0.025 anti-PT IgG IU of sample was used per well in 1% BSA-PBS (cat. no. 810033, MP Biomedicals, Solon, Ohio, USA) as described earlier [33], and were incubated on 200 ng of native purified PT (GSK, Rixensart, Belgium) on 96-well plates (art. no. 655061, Greiner Microlon, Frickenhausen, Germany). In-house controls of 0 and 650 anti-PT IgG IU mL^−1^ were included on each plate. The wells were treated with 100 µL of 6.5 M urea, 3.5 M urea, or PBS for 15 min. Both concentrations were previously demonstrated to separate avidity between individuals, without being either too mild or potent [33]. After anti-human IgG conjugate (0751-1002, KPL Inc., Maryland, USA), pnPP-substrate (cat. no. S0942, Sigma, Helsinki, Finland), and NaOH additions, the AI was counted from background reduced absorbance values, as a proportion of absorbance in urea-treated wells and absorbance in the PBS wells [33]. Several samples (*N* = 232) were first measured in duplicate wells to evaluate the average intra-assay variation in the AI values, resulting in a value of 4.1%. The inter-assay variance was studied in two replicate runs for 48 samples, resulting in a value of 9.3%.

### Statistics

AI values were analysed using IBM SPSS Statistics for Windows version 28.0 (IBM Corp., Armonk, NY, USA). AI values over 100% were converted to 100.0% for data analysis. Any samples with a lower absorbance measured from its PBS-treated well than the anti-PT IgG negative control well were excluded from further analysis. The differences in means between the groups at each time point were tested with ANOVA and Bonferroni corrections, and longitudinal analysis was done with paired T-tests. Two-sided *p*-values less than 0.05 were considered statistically significant. The correlation of AI to the overall anti-PT IgG IU mL^−1^ concentrations was calculated with the Pearson correlation coefficient.

## Results

### Avidity index of anti-PT antibodies in different age groups and between countries

**Figure 2.**
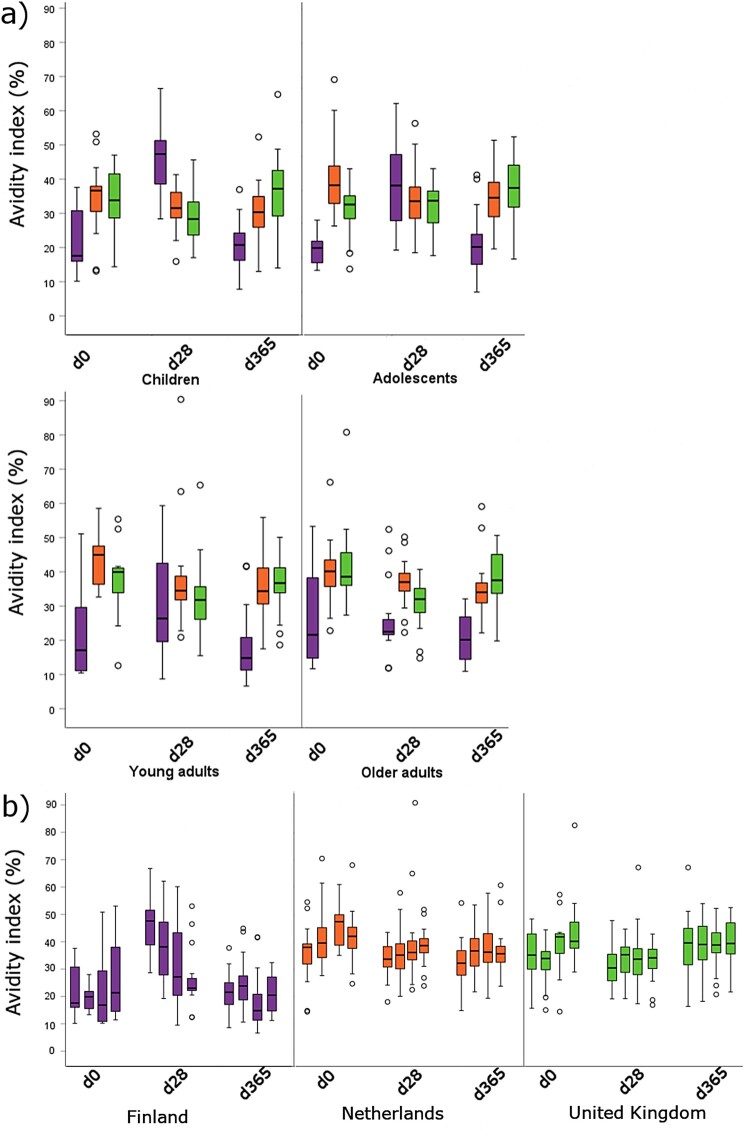
Avidity index measured with 6.5 M urea concentration from day 0, day 28, and day 365 after aP booster per age group (a) and country (b): Finland (purple), the Netherlands (orange), and the United Kingdom (green). In figure (b), the age groups are in the following order at each time point: children, adolescents, young adults, older adults. The box plots demonstrate the median, quartile range and 1.5 times the quartile range of inhibition of the study groups. o = values exceeding 1.5 times the interquartile range. The number of samples tested are specified in Table 1.

The development of the avidity index values for each age group per country at each time point for 6.5 M urea is presented in Figure 2, and for 3.5 M urea in Supplementary Figure 1, and the list of all statistically significant differences in avidity index is presented in Supplementary Table 1. The baseline AI was significantly lower in Finnish participants in comparison to those in the UK and the Netherlands across all age groups. In general, avidity remained stagnant or increased slightly in the Finnish and UK cohorts, whereas a decrease was noted among the Dutch cohorts. Children had lower baseline AI than older age groups. One month after the booster vaccination, AI was significantly higher in children compared to young and older adults in Finnish participants. One year after vaccination, significantly higher avidity was noted only in Finnish adolescents compared to young adults. There were no differences between age groups in the Netherlands or the UK after vaccination. In Finnish age cohorts, there was no change in avidity one year from baseline. In the Netherlands the decrease in avidity was significant in all age groups, whereas in the UK, there was a significant increase of avidity in children and adolescents.

The AI results between 6.5 and 3.5 M urea correlated closely within the countries (range of Pearson R 0.616–0.693 at day 0, 0.419–0.445 at one year). The correlation between the AI values obtained from 6.5 and 3.5 M urea treatments was found to be higher in subjects who had an increase in avidity (Pearson *R* = 0.700) compared to subjects with decreasing antibody avidity (Pearson *R* = 0.001).

### Development of avidity index based on pre-vaccination background

**Figure 3.**
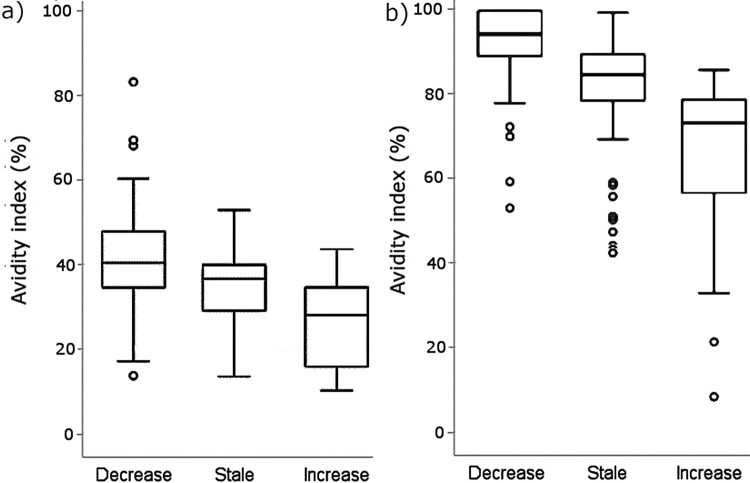
Avidity index before vaccination, measured with 6.5 M urea (a) and 3.5 M urea (b). Study participants have been distributed to categories based on having either at least a 10%-unit decrease, no increase or decrease (stale), or at least a 10%-unit increase in avidity one year after vaccination in comparison to baseline. The number of subjects in each category were 80, 70, and 52, respectively, for 6.5 M and 68, 82, and 47 for 3.5 M urea (all countries combined). o = values exceeding 1.5 times the interquartile range.

Those study participants with at least a 10% unit increase in AI at one year compared to baseline had significantly lower avidity pre-vaccination (*p* < 0.001) (Figure 3). Conversely, those with a decrease in AI had high pre-vaccination AI. Consequently, within the countries, the average values of AI for participants with low and high pre-vaccination avidity were noted to shift closer to each other, both at one month and one year after aP booster in comparison to baseline (Table 2). These trends were the strongest in the Netherlands.

**Table 2.** Geometric mean avidity index (%) in study participants at different time points in each study country based on either high or low avidity index before vaccination.

	6.5 M urea					3.5 M urea				
Country	Pre- vaccination avidity	*N*	Day 0	Day 28	Day 365	Pre- vaccination avidity	*N*	Day 0	Day 28	Day 365
FI	“Low” < 30%	29	17.7	31.9 ^a^	18.1	“Low” < 80%	25	54.8	79.9 ^a^	56.2
	“High” ≥ 30%	5	44.1	34.5 ^a^	20.5 ^a^	“High” ≥ 80%	7	86.3	76.7	60.7 ^a^
NL	“Low” < 30%	14	24.5	26.0 ^b^	24.4 ^b^	“Low” < 80%	11	58.9	80.4 ^b^	75.9 ^b^
	“High” ≥ 30%	85	40.4	33.8 ^a^	33.4 ^a^	“High” ≥ 80%	88	92.0	86.0 ^a^	82.8 ^a^
UK	“Low” < 30%	19	23.1	28.5 ^a/b^	32.2 ^a/b^	“Low” < 80%	35	67.9	78.6 ^a^	86.3 ^a^
	“High” ≥ 30%	45	39.2	33.0 ^a^	39.5	“High” ≥ 80%	27	86.7	81.5 ^a^	86.0

^a^ Statistical difference between time points d28 or d365 and d0, *p* < 0.05.

^b^ Statistical difference within a country between “Low” and “High,” *p* < 0.05.

AI was higher at baseline in adolescents who had been primed with DTwP in childhood, compared to Tdap-primed adolescents (Table 3). Both one month and one year after boosting, significantly higher avidities were noted in the DTwP group in comparison to Tdap-primed adolescents (*p* = 0.048) in the Netherlands. In Finland, no difference was observed between the groups after vaccination.

**Table 3.** Geometric mean avidity index of adolescents with different vaccination backgrounds.

Country	*N* (D0, 28, 365)	Vaccination Background	Avidity index 6.5 M urea			Avidity index 3.5 M urea		
			Day 0	Day 28	Day 365	Day 0	Day 28	Day 365
FI	6, 17, 15 4, 18, 17	aP wP	19.3 21.1	38.4 36.6	24.0 25.2	62.8 76.2	82.2 80.1	61.1 63.8
NL	23, 25, 25 15, 23, 22	aP wP	36.1 43.9^a^	31.6 36.3^a^	32.6 37.7^a^	85.9 94.8^a^	81.0 88.6^a^	82.3 88.0^a^

^a^ Statistical difference between wP and aP, *p* < 0.05.

### Relation between avidity and other antibody characteristics

**Figure 4.**
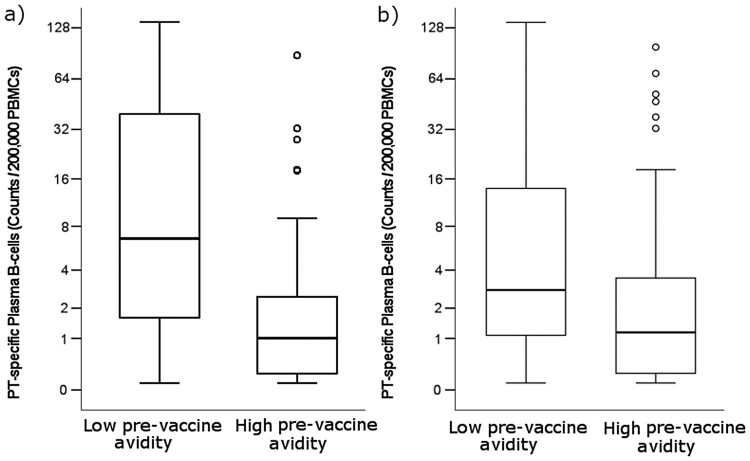
Study participants with lower baseline avidity-index (categorical-axis) defined as (a) < 30% AI for 6.5 M urea, or (b) < 80% AI for 3.5 M urea, had significantly higher plasma B cell counts to PT after vaccination (*p* < 0.001 6.5 M urea, *p* = 0.007 3.5 M urea). PBMC = peripheral blood mononuclear cells. The number of subjects in each category were 36 for low avidity and 94 for high avidity with 6.5 M urea, and 39 and 89 for 3.5 M urea (all countries combined). o = values exceeding 1.5 times the interquartile range.

If compared with data from previous studies with the same participants, within countries, avidity did not correlate with anti-PT IgG [7], PTNAs [27], or with numbers of plasma and memory B cells [28]. However, in an analysis with all countries combined, individuals with higher pre-vaccination AI had significantly lower plasma B cell counts (*p* < 0.001 6.5 M urea, *p* = 0.007 3.5 M urea) compared to those with low pre-vaccination AI (Figure 4). Out of these participants, those with high AI had half as much anti-PT IgG pre-vaccination (*p* < 0.001). These effects were individually noted as significant in Finland and the UK, but not in the Netherlands. Significantly higher avidity was also noted one year after vaccination among those participants (*N* = 35) with lower PT-specific memory B cell numbers (defined as less than five ELISPOTs per 200,000 peripheral blood mononuclear cells) pre-vaccination (*p* = 0.015).

**Figure 5.**
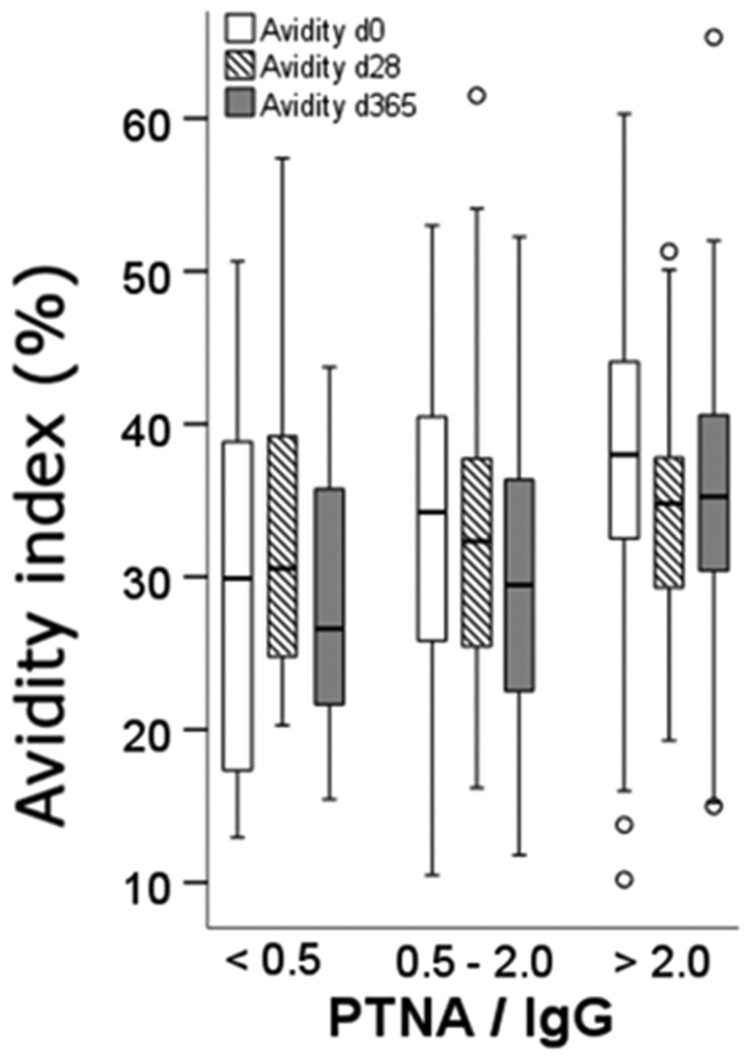
Study participants with higher pertussis toxin neutralizing antibody to anti-PT IgG ratio (PTNA/IgG) pre-vaccination (categorical-axis) had higher avidity index (y-axis) before vaccination (white plots) and one year after vaccination (grey plots), measured with 6.5 M urea, presented as a boxplot. No difference was noted at 28 days after vaccination (striped plots). The number of subjects, including all three study countries, were 138, 145, and 176 for d0, d28, and d365, respectively. o = values exceeding 1.5 times the interquartile range.

Participants whose anti-PT antibodies had relatively high neutralization capability in comparison to their overall anti-PT IgG concentrations pre-vaccination had significantly higher AI one year after vaccination (*p* = 0.021 6.5 M urea, *p* = 0.002 3.5 M urea) (Figure 5). As a definition, the ratio between PTNA and IgG describes how much neutralization a single IU of IgG causes [27]. For 3.5 M urea, the respective median values were 67.2%, 68.8%, and 80.2% for < 0.5, > 0.5 & < 2.0 and > 2.0 PTNA/IgG ratio categories. Similarly, participants with a high pre-vaccine PTNA/IgG ratio had higher pre-vaccine avidity (*p* = 0.022 6.5 M urea, *p* = 0.286 3.5 M urea). These trends were the strongest in the UK and the Netherlands, whereas in Finland there were no notable differences between the categories. This may have been due to a relatively low number of samples with a high PTNA/IgG ratio in the Finnish cohort (28%), in comparison to the Netherlands (56%) and the UK (53%).

## Discussion

The common premise is that antibody avidity increases after vaccination and with repeated exposure to the target antigen [16,26,32,34,35]. However, this might not always be the case, as demonstrated by this study: Those subjects with high existing AI had on average no increase in AI after Tdap booster. Vaccination increased AI to PT especially in subjects with relatively low pre-vaccination AI. Thereafter, booster vaccinations seems particularly beneficial regarding antibody avidity development to those individuals with relatively low antibody avidity levels, and it remains to be studied whether an additional, closely timed booster dose would further improve anti-PT avidity in these individuals. No connection was found between avidity development and baseline antibody concentrations or memory B cell counts. In this same vaccine cohort, individuals with higher memory B cell counts pre-Tdap booster also had elevated B cell counts post-vaccination [23]. At least for PT, there is an additional proliferation of B cells in subjects with low AI pre-vaccination (Figure 4). Moreover, in participants with high avidity pre-vaccination, a weak correlation between antibody avidities from 6.5 and 3.5 M urea treatments was observed one year after vaccination. It could be deduced that in these individuals, booster vaccination induces a different profile of avidity compared to existing antibody avidity. Together, these findings indicate that the existing antibodies with high avidity affect various vaccination responses to PT. The affinity development in the case of existing immunity may be further related to differences in the epitope specificity of anti-PT antibodies after Tdap vaccination and natural infection [26].

Children, having received multiple vaccine doses and thus repeated encounters with (detoxified) PT more recently than adults, likely possess greater immunological memory and avidity. Although the original antigenic sin phenomenon, which refers to the tendency of the immune system to preferentially utilize immunological memory based on a first encounter with an antigen, is mostly observed with viral diseases [36,37], the differences in the structure between chemically detoxified PT in the vaccines and PT from natural infection may lead to inferior avidity in children towards native PT, and respectively possibly to inferior protection [38]. Frequent Tdap dosing may amplify this effect, at least concerning the avidity of anti-PT IgG antibodies. Whereas age-based trends were visible in Finland, particularly as higher avidity in adolescents compared to young adults, it seems that at an individual level, the prior level of avidity has a higher influence on AI than age. Differences in pertussis incidence and in laboratory methods to evaluate incidence, as well as in in vaccine coverage between the study countries and between age groups [29,39] (Figure 1) may reflect existing immunity levels, affecting the different avidity development trends. A Japanese seroprevalence study [40] found no age-related differences in the AI to PT, similar to the baseline observation in this study. A shorter time since the latest vaccination has been shown to positively contribute to avidity development towards PT [33]. In this study, 4–11 years have passed since the latest vaccinations for children and adolescents, possible reducing the boosting effect than what was observed for children younger than 5 years of age who received Tdap [33]. Whereas the general induction trend of anti-PT IgG, PT-specific B-cells, and PTNAs after vaccination was similar in all age groups and between countries [7,27,28], avidity development seems more background dependent.

Based on the acquired data, avidity after vaccination varies based on country, age group, existing avidity, and time point. Higher avidities were previously noted in aP-primed children compared to wP-primed at four years of age, one month after an aP booster [41]. In this study, the avidity of aP-primed adolescents was slightly higher in Finland, but significantly lower in the Netherlands, in comparison to the wP group one month after vaccination. Differences noted at one month may gradually turn in favour of wP priming in the long term. Likewise, pre-vaccine avidity had remained higher in wP-primed adolescents in both Finland and the Netherlands. Longer evaluation periods than one month are clearly needed to confirm the direction of avidity development. The divergent vaccination backgrounds, number of doses received, and the age range in the adolescent age group may influence the results [7, 22].

PT is an exotoxin with many biological activities *in vivo*. Evaluating the ability of vaccination-induced PT-specific antibodies to prevent these activities of PT requires functional evidence since antibody quantity alone is not enough to estimate protection. Different aspects have been studied in the field, including the neutralization of leukocytosis-promoting activity and enzymatic activity, opsonophagocytosis, and the binding characteristics [26]. All these aspects contribute to the effectiveness of the antibodies to clear out the toxin.

It is of further interest to determine if these characteristics correlate with each other to identify the best tests to evaluate antibody maturation, protection, and vaccination efficacy. Different methods provide distinct information, even among monoclonal antibodies (targeting the same subunits of PT); some antibody clones excel in PTNA capabilities yet vary in affinity [42]. Since PTNA function mainly relates to neutralizing the enzymatic subunit 1 of PT, alternating antibody affinity to other subunits may create a weak observed correlation between the two. In that sense the connection between PT neutralization and higher avidity antibodies at baseline found in this study was an unexpected finding. As a side note, this connection was only found in the analysis model if the overall anti-PT IgG concentrations were considered [27,40]. The noted relationship between PTNA/anti-PT IgG ratio and avidity may also be a closer reflection of age-related antibody maturation, as infants have significantly lower PTNA/IgG ratios in comparison to adults [43]. The low sample size for country specific age groups in this study can limit the statistical power for the performed analyses, and further studies with larger age cohorts are needed to create a more reliable estimate of the effect between different age groups. Having high avidity and neutralizing antibodies before vaccination seems to be a predictor for sustaining relatively higher avidity antibodies up to one year after vaccination, despite a notable decrease in both avidity (Figure 3 and 5) and PTNA/anti-PT IgG ratio [27]. Similarly, the quantities of anti-PT IgG and PTNA after booster are positively influenced by a high existing antibody concentration [27,43] and by low levels of IFN-γ, IL-2, IL-10, and IL-17A [44]. Thus many immunological factors influence the anti-PT IgG responses, including hypothetically avidity as well. All in all, the functionality of the Tdap booster vaccination-induced antibodies seems weaker than that of antibodies detected at baseline, which have remained from long-induced immunity.

Functional antibody assays are often laborious, require extensive amounts of serum and handling of eukaryotic cell lines [26]. From a practical point of view, avidity measurement is a rather scalable assay, faster, less laborious, and uses serum, which is also easier to collect in comparison to peripheral blood mononuclear cells required to perform B cell-related ELISPOTs or flow cytometry [28]. However, as a standardized assay for studying avidity against pertussis antigens is not established, comparisons to earlier studies is difficult. Although this study found no notable differences in the development of avidity between the different urea concentrations, we encourage future research to include a range of chaotropic agent concentrations that best reflect the maturation of antibodies. Different avidities are obtained with varying assay conditions, and selecting an appropriate chaotropic agent and its concentration is critical when AI is evaluated [26]. Although samples were diluted to the same antibody concentration, differences in antibody concentrations might still indirectly affect avidity assays. In addition, this study is focused solely on the pertussis toxin component of the Tdap3-IPV vaccine. It remains of great interest to evaluate the long-term development of AI after the booster vaccination by other pertussis vaccines and to other vaccine components or protein lysate of *B. pertussis*. Developing functional methods remains an essential task, since no established correlate of protection exists for pertussis. For now, providing booster vaccinations seems particularly beneficial regarding antibody avidity development to those individuals with relatively low antibody avidity levels, and to sustain high avidity in individuals with already relatively high avidity levels.

## Supplementary Material

Supplementary figure 1.tif

Supplementary Table 1.docx

## Data Availability

Individual participant data that underlie the results reported in this article have been de-identified and deposited in the central database of the PERISCOPE Consortium and can be accessed by a request to the PERISCOPE management team.
