# Supplementary figures and images for: Prior immunological memory to pertussis toxin affects the avidity development of anti-PT IgG antibodies after acellular pertussis booster vaccination
Source: Emerg Microbes Infect. 2025 Aug 13;14(1):2547720. doi: 10.1080/22221751.2025.2547720 (PMC12406325; doi:10.1080/22221751.2025.2547720)

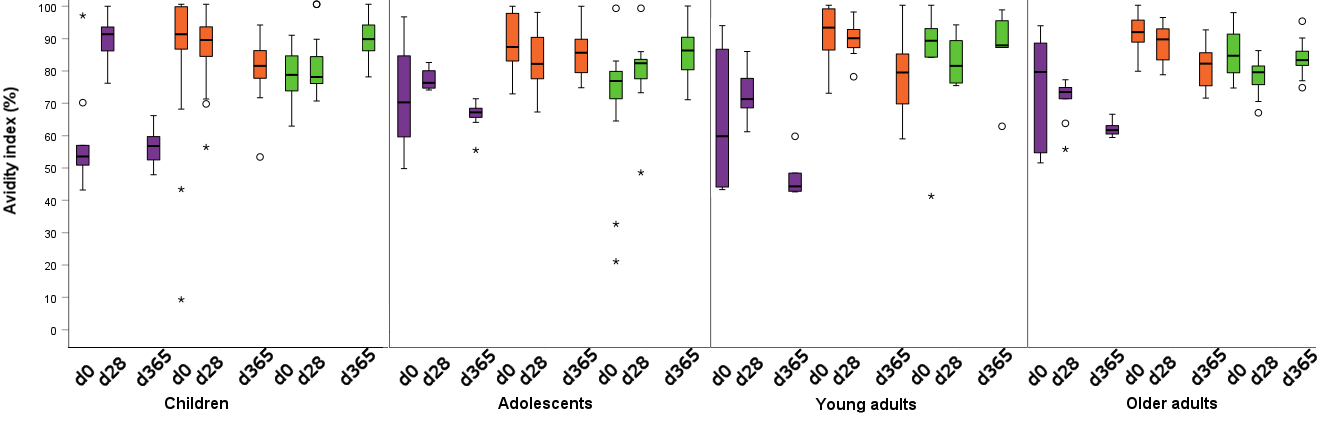

Supplement: Supplementary figure 1.tif [file TEMI_A_2547720_SM9390.tif]
