# Supplementary material for: Prior immunological memory to pertussis toxin affects the avidity development of anti-PT IgG antibodies after acellular pertussis booster vaccination
Source: Emerg Microbes Infect. 2025 Aug 13;14(1):2547720. doi: 10.1080/22221751.2025.2547720 (PMC12406325; doi:10.1080/22221751.2025.2547720)
Supplement: Supplementary Table 1.docx [file TEMI_A_2547720_SM9389.docx]

**Supplementary Table 1.** Statistically significant differences in avidity index between age groups, countries, and longitudinally within age groups

| Cohort | ^a^ Higher >  or lower < | Comparison cohort | 6.5 M / 3.5 M urea | Time point | p-value |
| --- | --- | --- | --- | --- | --- |
| ^b^ FI Children | > | FI Adolescents,  Young adults, Older adults | 6.5 & 3.5 | d28 | <0.033 |
| FI Children | < | NL Children | 6.5 & 3.5 | d0, d365 | <0.001 |
| FI Children | > | NL Children | 6.5 | d28 | <0.001 |
| FI Children | < | UK Children | 6.5 | d0 | 0.004 |
| FI Children | > | UK Children | 6.5 & 3.5 | d28 | <0.001 |
| FI Children | < | UK Children | 6.5 & 3.5 | d365 | <0.001 |
| FI Children d28 | > | FI Children | 6.5 & 3.5 | d0, d365 | <0.001 |
| NL Children | < | UK Children | 3.5 | d28 | 0.006 |
| NL Children | < | UK Children | 6.5 & 3.5 | d365 | <0.008 |
| NL Children d28,365 | < | NL Children | 6.5 | d0 | <0.014 |
| NL Children d365 | < | NL Children | 3.5 | d28 | 0.016 |
| UK Children d28  UK Children d365  UK Children d365 | <  >  > | UK Children  UK Children  UK Children | 6.5  3.5  6.5 & 3.5 | d0  d0  d28 | 0.049  <0.001  <0.002 |
| FI Adolescents | > | Older adults | 6.5 & 3.5 | d28 | <0.002 |
| FI Adolescents | > | Young adults | 6.5 & 3.5 | d365 | <0.012 |
| FI Adolescents | < | NL Adolescents | 6.5 & 3.5 | d0, d365 | <0.001 |
| FI Adolescents | < | UK Adolescents | 6.5 | d0 | <0.001 |
| FI Adolescents | < | UK Adolescents | 6.5 & 3.5 | d365 | <0.001 |
| FI Adolescents d28 | > | FI Adolescents | 6.5 & 3.5 | d0, d365 | <0.005 |
| NL Adolescents | > | UK Adolescents | 3.5 | d0 | <0.001 |
| NL Adolescents d28 | < | NL Adolescents | 6.5 & 3.5 | d0 | <0.001 |
| NL Adolescents 365 | < | NL Adolescents | 6.5 | d0 | <0.001 |
| NL Adolescents 365 | > | NL Adolescents | 6.5 | d28 | 0.024 |
| UK Adolescents d365 | > | UK Adolescents | 6.5 & 3.5 | d0 | <0.002 |
| UK Adolescents d365 | > | UK Adolescents | 3.5 | d28 | 0.035 |
| UK Adolescents d28 | > | UK Adolescents | 3.5 | d0 | 0.037 |
| FI Young adults | < | NL Young adults | 6.5 & 3.5 | d0 | <0.021 |
| FI Young adults | < | NL Young adults | 3.5 | d28 | <0.001 |
| FI Young adults | < | NL, UK Young adults | 6.5 & 3.5 | d365 | <0.001 |
| FI Young adults d365 | < | FI Young adults | 6.5 & 3.5 | d28 | <0.005 |
| NL Young adults | < | UK Young adults | 3.5 | d28 | <0.001 |
| NL Young adults d28 | < | NL Young adults | 6.5 & 3.5 | d0 | <0.002 |
| NL Young adults d365 | < | NL Young adults | 6.5 | d0 | 0.004 |
| UK Young adults d365 | > | UK Young adults | 6.5 & 3.5 | d28 | <0.038 |
| FI Older adults | < | UK, NL Older adults | 6.5 & 3.5 | d0, d28, d365 | <0.049 |
| FI Older adults 365 | < | FI Older adults | 6.5 & 3.5 | d28 | <0.033 |
| FI Older adults 365 | < | FI Older adults | 3.5 | d0 | 0.045 |
| NL Older adults | > | NL Children, Adolescents | 6.5 | d0 | <0.038 |
| NL Older adults | > | UK Older adults | 6.5 & 3.5 | d28 | <0.036 |
| NL Older adults d0 | > | NL Older adults | 6.5 & 3.5 | d28, d365 | <0.016 |
| NL Older adults d365 | < | NL Older adults | 6.5 & 3.5 | d28 | <0.029 |
| UK Older adults d28 | < | UK Older adults | 6.5 & 3.5 | d0, d365 | <0.007 |

^a^ Indication of which, “Cohort” or the “Comparison cohort” was statistically significantly higher or lower than the other

^b^ FI = Finland, NL = The Netherlands, UK = The United Kingdom
